# P-1869. Utilizing Large Language Models for Enhanced Decision Support in Travel Medicine Clinic: our experience at Mayo Clinic

**DOI:** 10.1093/ofid/ofae631.2030

**Published:** 2025-01-29

**Authors:** John C O’Horo, Haris Akhtar, Vish Anantraman, Muhammad Ammar, Juro Gottweis, Douglas Challener

**Affiliations:** Mayo Clinic, Rochester, Minnesota; Mayo Clinic Rochester, Rochester, Minnesota; Mayo Clinic Rochester, Rochester, Minnesota; Google, Arlington, Virginia; Google, Arlington, Virginia; Mayo Clinic, Rochester, Minnesota

## Abstract

**Background:**

The integration of Generative AI (GAI) into healthcare systems is increasingly recognized for its potential to transform patient management. The primary aim of this research was to evaluate and quantify the performance of large language models (LLMs) in generating actionable travel medicine advice.

Architectural design of the Travel Clinic LLM project.

**Figure:**
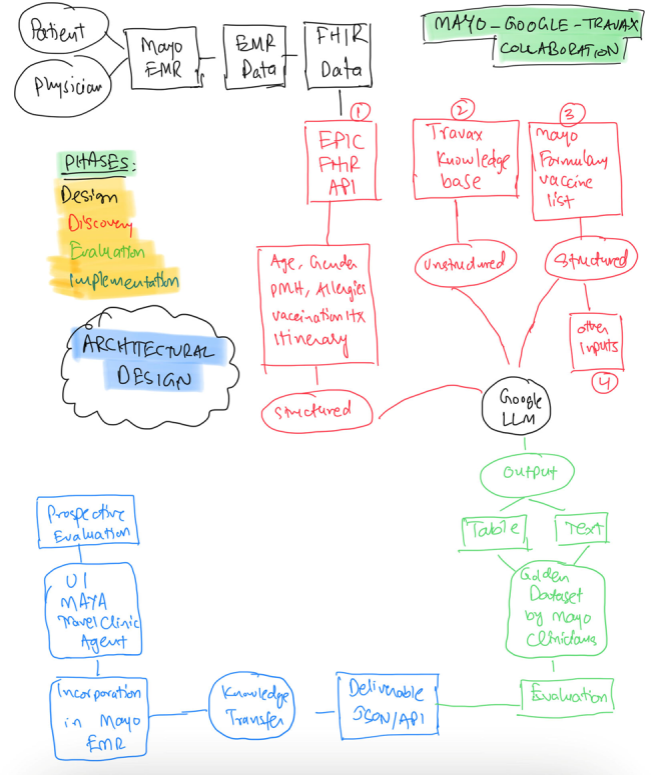

Four phases of Discovery, Design, Evaluation, and Implementation/Deployment.

**Methods:**

This study utilized two iterative phases of evaluation. In the initial phase, LLMs were prompted with detailed clinical scenarios including demographic data, medical and immunization histories, and specific travel plans. These prompts were designed to mimic typical inquiries encountered in travel consultations. The LLMs' initial responses were generated using the CDC’s Yellow Book as a foundational knowledge base. In the subsequent phase, the prompts were refined for greater specificity and clarity, and the knowledge base was enhanced by transitioning to Travax’s Travelers’ Health database. Additional structured data inputs included an exhaustive list of vaccines from our pharmacy formulary and a detailed table of vaccine contraindications. The responses were evaluated and scored by ID clinicians from the Mayo Clinic.

**Results:**

Initial findings after first iteration revealed limited efficacy with recall at 23.9%, an F1 score of 38.6%, accuracy also at 23.9%, and precision maintained at 100%, utilizing the CDC's Yellow Book. With the implementation of Travax and refined prompting techniques, preliminary results suggest a notable improvement in the quality of responses, though detailed scoring is presently underway.

Improvements in the LLM’s performance can be attributed to several key adjustments: the adoption of a more comprehensive knowledge base, refined prompt engineering, and the incorporation of structured data to support more accurate and detailed recommendations. The collaborative engagement of Mayo Clinic with Google and Travax facilitated a synergistic approach to optimizing the AI model's utility and integration. Future plans include embedding the LLM into our EMR system.

**Conclusion:**

The findings from this study highlight the significance of strategic collaborations between large healthcare centers, IT industry, and specialized knowledge database firms in effectively harnessing GAI for clinical use.

**Disclosures:**

All Authors: No reported disclosures

